# Endovascular Interventions for Peripheral Arterial Disease in Saudi Patients: A Cross-Sectional Study Assessing Efficacy and Economic Impact

**DOI:** 10.7759/cureus.49225

**Published:** 2023-11-22

**Authors:** Abdulsalam Aleid, Rahaf Alghareeb, Eyad Omar, Ayman S Almukhlifi, Abdulmohsen Mohammad, Ali A Alqarni, Yazid Binobaiad, Abbas Al Mutair, Mohammed Alessa, Loai Albinsaad

**Affiliations:** 1 Department of Surgery, King Faisal University, Al-Ahsa, SAU; 2 College of Medicine, King Faisal University, Al-Ahsa, SAU; 3 College of Medicine, Jazan University, Jazan, SAU; 4 College of Medicine, Taibah University, Madinah, SAU; 5 College of Medicine, King Khalid University, Abha, SAU; 6 Research Center, Almoosa Specialist Hospital, Al-Ahsa, SAU

**Keywords:** cost-effectiveness analysis, effectiveness assessment, saudi patients, endovascular interventions, peripheral arterial disease

## Abstract

Introduction

Peripheral Arterial Disease (PAD) is characterized by arterial narrowing or blockage, causing pain and reduced quality of life. Endovascular interventions, like angioplasty and stenting, offer less invasive treatment options with potential benefits. This study, conducted in the Al-Ahsa region of Saudi Arabia from January to August 2023, aims to assess the impact of these interventions on PAD management in Saudi patients. Specifically, we aim to evaluate their effectiveness in improving clinical outcomes, such as symptom relief and quality of life, and their cost-effectiveness in the Saudi healthcare system. By addressing these objectives, our research provides evidence to support informed clinical decisions and healthcare policy in Saudi Arabia, ultimately enhancing patient care.

Methods

In this study, a cross-sectional design was used to assess the impact of endovascular interventions on PAD management in Saudi patients in the Al-Ahsa region. Data collection took place from January to August 2023. The study focused on a sample of 385 or more Saudi patients who had undergone endovascular interventions. Inclusion criteria involved diagnosed PAD patients who had received these interventions, while non-Saudi patients and those without PAD or interventions were excluded. Data was collected through an online questionnaire distributed in hospitals. The study considered demographic and clinical/economic variables to evaluate intervention effectiveness and cost-effectiveness.

Results

The findings of this study emphasize the significance of variables such as gender, education level, employment status, and geographic location in shaping perceptions concerning the effectiveness and cost-effectiveness of endovascular interventions for the management of PAD. Participants in the study reported substantial improvements in symptom relief, quality of life, and daily activities following endovascular interventions. Moreover, the study revealed divergent perceptions regarding the cost-effectiveness of these interventions among participants.

Conclusion

This study highlights a positive association between the use of endovascular interventions and improved clinical outcomes in Saudi patients diagnosed with PAD. The results of this research indicate that endovascular interventions are not only more cost-effective when compared to alternative treatment modalities for PAD management but also lead to significant enhancements in symptom relief, quality of life, and daily activities among patients. The implications of these findings for the Saudi healthcare system are substantial, underscoring the importance of evidence-based decisions in the adoption of endovascular interventions for PAD management.

## Introduction

In the context of modern medicine, Peripheral Arterial Disease (PAD) stands as a widespread and significant condition, characterized by the narrowing or blockage of peripheral arteries, ultimately leading to a reduction in blood flow and tissue oxygenation. The etiology of PAD is often rooted in systemic atherosclerotic processes, which, if left unaddressed, can result in life-altering vascular events such as stroke and myocardial infarction [1]. The global impact of PAD is substantial, with an estimated 200 million individuals affected worldwide [2]. In the specific context of Saudi Arabia, recent research has unveiled a prevalence rate of approximately 11.7% (n=56) of 471 patients were recruited, with projections suggesting a significant increase as the population ages and confronts various risk factors, including hypertension, diabetes, smoking, lipid disorders, cerebrovascular events, and coronary artery disease [3].

The clinical presentation of PAD is highly variable, ranging from asymptomatic cases to individuals experiencing intermittent pain or discomfort in the affected limb, commonly known as "claudication." In more severe instances, PAD can manifest as non-healing wounds and ulcers, with the severity of symptoms contingent on the location and extent of arterial blockages. Left untreated, PAD can lead to severe complications, including the risk of amputation and even death. Conditions such as Chronic Limb Ischemia (CLI) may emerge when the obstruction becomes profound, potentially resulting in ulceration, gangrene, or rest pain. Acute Limb Ischemia (ALI), on the other hand, may occur if an atherosclerotic plaque ruptures in a peripheral artery, leading to the sudden onset of symptoms like pain, pallor, sensory deficits (paresthesia), or paralysis due to the abrupt limitation of blood supply. Such clinical manifestations significantly impact the quality of life, often causing functional impairment and disability in a substantial number of patients [4-7].

Moreover, beyond the profound health implications, PAD imposes a substantial economic burden on healthcare systems and patients alike, particularly in the management of advanced disease. Therefore, the cost-effectiveness of interventions in PAD management is a crucial consideration [8-10]. Notably, endovascular interventions, encompassing techniques like angioplasty and stenting, have emerged as promising strategies for treating PAD. These interventions involve minimally invasive procedures designed to restore blood flow and alleviate symptoms by addressing arterial blockages through balloons, stents, or other devices. Despite their potential, the effectiveness and cost-effectiveness of endovascular interventions remain topics of debate [11].

Several studies have explored their effectiveness, with some indicating benefits, such as improved walking distance and a lower risk of major amputation [12-14]. However, these studies have limitations, including small sample sizes and short follow-up periods, leaving questions about their applicability and outcomes in specific contexts, such as Saudi Arabia. To date, the effectiveness and cost-effectiveness of endovascular interventions in the Saudi healthcare system remain unclear. It is within this knowledge gap that our cross-sectional study takes shape, aiming to investigate the impact of endovascular interventions on the management and cost of PAD among Saudi patients. We hypothesize a positive correlation between the use of endovascular interventions and improved clinical outcomes in Saudi patients with PAD. Additionally, we postulate that these interventions offer enhanced cost-effectiveness when compared to alternative treatment modalities within the Saudi healthcare system.

This research endeavor holds both medical and economic significance, offering an opportunity to augment our understanding of PAD management and contribute valuable insights for healthcare practitioners and policymakers alike. The objectives of this study are twofold: to assess the effectiveness of endovascular interventions in improving clinical outcomes, specifically symptom relief and quality of life among Saudi patients with PAD, and to evaluate the cost-effectiveness of these interventions compared to other treatment modalities in the Saudi healthcare system. By addressing these objectives, our research aims to bridge the existing knowledge gap, ultimately impacting the management and healthcare delivery for PAD in the Saudi context.

## Materials and methods

Study design

In the present study, we adopt a cross-sectional design, which aligns with our research objectives by allowing us to explore the impact of endovascular interventions on Peripheral Arterial Disease (PAD) management among Saudi patients. The Al-Ahsa region in Saudi Arabia serves as the study area, and data collection took place from June to August 2023.

Study population

Our study focuses on a representative sample of Saudi patients with PAD who have undergone endovascular interventions. These individuals are integral to our investigation as they provide valuable insights into the effectiveness and cost-effectiveness of these interventions in the Saudi healthcare context.

Sample size and sampling technique

Considering Saudi Arabia's population. We determined that a sample size of 385 or more measurements/surveys would be necessary to achieve a confidence level of 95% within ±5% of the measured/surveyed value.

Inclusion criteria

Inclusion criteria were defined to ensure the relevance and applicability of our study. In this context, we included Saudi patients who were diagnosed with PAD and had undergone endovascular interventions.

Exclusion criteria

Exclusion criteria were established to maintain the study's specificity. Consequently, non-Saudi patients and individuals who had not been diagnosed with PAD or undergone endovascular interventions were excluded from the sample.

Data collection tools

Data collection was facilitated through the utilization of an online questionnaire, designed to gather information from the patients. Additionally, this questionnaire was distributed in hospitals, ensuring a comprehensive and diverse pool of responses.

Study variables

Our study encompasses both independent and dependent variables. Independent variables comprise demographic data, including age, gender, education level, employment status, city of residence, and geographic location. Dependent variables include clinical and economic metrics, specifically related to symptom relief, quality of life measures, cost data, and healthcare resource utilization.

Ethical considerations

To uphold the highest ethical standards, strict confidentiality was maintained throughout the data collection process. No information that could potentially pose ethical issues, such as participant names, was used. Ethical approval for this study was granted by the King Faisal University Research Ethics Committee, further underlining our commitment to ethical practices. The IRB approval number is KFU-REC-2023-SEP-ETHICS1199.

Statistical analyses

For the comprehensive analysis of data, we employed SPSS version 28.0 (IBM Corp., Armonk, USA), conducting a range of statistical analyses. Descriptive statistics were employed to summarize demographic characteristics, symptom profiles, and perceptions related to the effectiveness and cost-effectiveness of endovascular interventions in PAD management. Multivariate analysis, including logistic regression and linear regression models, was conducted to identify demographic factors predicting effectiveness and cost-effectiveness. Odds ratios (OR) with 95% confidence intervals (CI) were reported. Cronbach's alpha coefficient was calculated to assess the internal consistency of Likert scale sections. A significance level of p < 0.05 was considered statistically significant. These analytical approaches provided a robust framework for comprehensively exploring the factors influencing participants' perceptions of endovascular interventions in PAD management.

## Results

Demographic characteristics

The demographic characteristics of the participants are summarized in Table 1. The majority of participants were female 68.9% (n=936) and had a bachelor's degree 65.6% (n=891). Employed full-time participants constituted the largest employment group 43.7% (n=594), and the Western province was the most common city of residence 19.2% (n=261). Most participants were from urban areas 85.4% (n=1161).

**Table 1:** Demographic characteristics. The table presents the distribution of participants' age, gender, education level, employment status, city of residence, and geographic location.

		Count	N %
Age	24-18	495	36.4%
	34-25	189	13.9%
	44-35	279	20.5%
	54-45	288	21.2%
	55 - 64	63	4.6%
	Under 18	27	2.0%
	Above 65	18	1.3%
Gender	Female	936	68.9%
	Male	423	31.1%
Education level	High school or less	306	22.5%
	Diploma	90	6.6%
	Bachelor's degree	891	65.6%
	Doctorate or higher	27	2.0%
	Master's degree	45	3.3%
Employment Status	Student	387	28.5%
	Unemployed	261	19.2%
	Retired	90	6.6%
	Employed full-time	594	43.7%
	Employed part-time	27	2.0%
City of residence	Western province	261	19.2%
	Middle Province	72	5.3%
	Eastern Province	540	39.7%
	South Province	486	35.8%
Geographic Location:	Urban	1161	85.4%
	Rural	99	7.3%
	Suburban	99	7.3%

Table 2 presents responses related to the perceived cost-effectiveness of endovascular interventions. While 6.6% (n=90) of participants reported being diagnosed with PAD, most participants had not undergone endovascular interventions 94.7% (n=1287). Common symptoms included leg pain or cramping during physical activity 10% (n=150) and coldness or numbness in the legs or feet 2.6% (n=36). A considerable proportion of participants reported a significant improvement in symptoms and quality of life 11.9% (n=162) after the intervention.

**Table 2:** General questions regarding the cost-effectiveness of endovascular surgery in treatment of PAD This table displays participants' responses related to their PAD diagnosis, symptoms, quality of life, and perceptions of endovascular intervention's cost-effectiveness. PAD: Peripheral Arterial Disease

		Count	N %
Have you been diagnosed with peripheral arterial disease (PAD)?	No	1269	93.4%
	Yes	90	6.6%
How long have you been experiencing symptoms related to PAD?	From 6 months to 1 year	63	4.6%
	Less than 6 months	180	13.2%
	More than 3 years	9	0.7%
	From 1 to 3 years	18	1.3%
Have you previously undergone any endovascular interventions for PAD management?	No	1287	94.7%
	Yes	72	5.3%
Which of the following symptoms are you currently experiencing? (Select all that apply)	Leg pain or cramping during physical activity	150	10%
	Leg pain or cramping at rest	40	3.0%
	Coldness or numbness in your legs or feet	36	2.6%
	Coldness or numbness in your legs or feet	18	1.3%
	Weakness or fatigue in your legs	18	1.3%
How would you rate your overall quality of life before the intervention?	Good	81	6.0%
	Poor	27	2.0%
	Fair	108	7.9%
	Excellent	162	11.9%
Have you noticed any improvement in your symptoms and quality of life after the endovascular intervention?	No improvement	27	2.0%
	Yes, slight improvement	36	2.6%
	Yes, significant improvement	162	11.9%
	Yes, moderate improvement	45	3.3%
Which of the following treatment options were discussed with you before the endovascular intervention? (Select all that apply)	Surgical intervention,	65	5.3%
	Medication therapy	150	10.0%
	Lifestyle modifications (exercise, diet changes)	153	10.9%
In your opinion, do you believe that the endovascular intervention was a cost-effective treatment option for PAD management?	Unsure	153	11.3%
	No	72	5.3%
	Yes	108	7.9%
Did you experience any complications or adverse events related to the endovascular intervention?	No	297	21.9%
	Yes	54	4.0%
How satisfied are you with the overall outcome of the endovascular intervention?	Very satisfied	99	7.3%
	Satisfied	81	6.0%
	Neutral	108	7.9%

Table 3 provides insights into the effectiveness of endovascular interventions. On average, participants reported a moderate level of symptom relief (mean = 5 ± 1.2).

**Table 3:** Effectiveness of endovascular intervention (Mean ± SD)

	Mean ± SD
On a scale of 1 to 10, rate the level of symptom relief you experienced after the endovascular intervention, with 1 being no relief and 10 being complete relief.	5 ± 1.2

Table 4 reports that a substantial proportion of participants reported improvements in various aspects, including the ability to perform daily activities 10.6% (n=144), wound healing or ulcer improvement 9.9% (n=135), and reduction in leg pain or cramping during physical activity 7.9% (n=108). Furthermore, 8.6% (n=117) of participants reported moderate improvement in their overall quality of life post-intervention.

**Table 4:** Effectiveness of endovascular intervention The table summarizes participants' reported symptom relief, improvements in daily activities, wound healing, pain reduction, and overall quality of life post-endovascular intervention. PAD: Peripheral Arterial Disease

		Count	N %
Have you noticed any improvement in your ability to perform daily activities (e.g., walking, climbing stairs) after the endovascular intervention?	Yes, slight improvement	45	3.3%
	Yes, significant improvement	99	7.3%
	Yes, moderate improvement	144	10.6%
Are you currently experiencing any wound healing or ulcer improvement since the endovascular intervention?	Yes, slight improvement	63	4.6%
	Yes, significant improvement	72	5.3%
	Yes, moderate improvement	135	9.9%
How often do you experience leg pain or cramping during physical activity after the endovascular intervention?	Never	72	5.3%
	Frequently	63	4.6%
	Always	9	0.7%
	Occasionally	108	7.9%
	Rarely	63	4.6%
Has your overall quality of life improved since the endovascular intervention?	Yes, moderately improved	117	8.6%
	Yes, significantly improved	72	5.3%
	Yes, slightly improved	99	7.3%
Are you able to walk longer distances without experiencing leg pain or cramping after the endovascular intervention?	Yes, significantly longer distances	99	7.3%
	Yes, slightly longer distances	81	6.0%
	Yes, moderately longer distances	126	9.3%
Have you noticed any improvement in the temperature and sensation in your legs and feet since the endovascular intervention?	Yes, slight improvement	54	4.0%
	Yes, significant improvement	108	7.9%
	Yes, moderate improvement	126	9.3%
How often do you experience weakness or fatigue in your legs after the endovascular intervention?	Never	54	4.0%
	Frequently	36	2.6%
	Occasionally	117	8.6%
	Rarely	90	6.6%
Would you recommend endovascular intervention as a treatment option to other patients with PAD?	No, probably not	45	3.3%
	Undecided	45	3.3%
	Yes, definitely	117	8.6%
	Yes, probably	108	7.9%
How likely are you to undergo endovascular intervention again if needed in the future?	Unlikely	18	1.3%
	Neutral	81	6.0%
	Very likely	63	4.6%
	Likely	108	7.9%
	Very unlikely	27	2.0%

The results of a Likert analysis assessing the effectiveness are detailed in Table 5. Participants reported moderate levels of improvement in various aspects related to PAD management, such as the ability to perform daily activities (mean = 3.72; n=1359), wound healing or ulcer improvement (mean = 3.46; n=1359), and reduction in leg pain or cramping (mean = 3.96; n=1359).

**Table 5:** Likert analysis of the effectiveness This table presents the mean ratings and standard deviations for participants' perceived improvements in various aspects following the endovascular intervention. PAD: Peripheral Arterial Disease

	N	Minimum	Maximum	Mean	SD
Have you noticed any improvement in your ability to perform daily activities (e.g., walking, climbing stairs) after the endovascular intervention?	1359	1	5	3.72	1.319
Are you currently experiencing any wound healing or ulcer improvement since the endovascular intervention?	1359	1	5	3.46	1.228
How often do you experience leg pain or cramping during physical activity after the endovascular intervention?	1359	1	5	3.96	1.064
Has your overall quality of life improved since the endovascular intervention?	1359	1	5	3.24	1.674
Are you able to walk longer distances without experiencing leg pain or cramping after the endovascular intervention?	1359	1	5	3.66	1.201
Have you noticed any improvement in the temperature and sensation in your legs and feet since the endovascular intervention?	1359	1	5	3.63	0.134
How often do you experience weakness or fatigue in your legs after the endovascular intervention?	1359	1	5	3.94	0.889
Would you recommend endovascular intervention as a treatment option to other patients with PAD?	1359	1	5	1.05	0.049
How likely are you to undergo endovascular intervention again if needed in the future?	1359	1	5	3.72	1.319

The multivariate analysis aimed at identifying demographic factors predicting effectiveness is presented in Table 6. Gender significantly influenced effectiveness (OR = 1.23, 95% CI = 1.20 - 1.98, p < 0.001). Education level was also significant (p < 0.001), with participants holding a master's degree reporting lower effectiveness (OR = 0.60, 95% CI = 0.42 - 0.85).

**Table 6:** Multivariate analysis of demographic factors predicting the effectiveness. The table outlines odds ratios and confidence intervals for factors influencing the effectiveness of endovascular interventions, including gender, education, and employment status.

Variable	Odds Ratio (OR)	95% Confidence Interval (CI)	P-value
Gender (Reference: Female)	1.23	1.20 - 1.98	<0.001
Education level			<0.001
- High school or less	1 (Reference)		
- Diploma	1.28	0.68 - 1.25	
- Bachelor's degree	1.95	0.61 - 1.01	
- Master's degree	0.60	0.42 - 0.85	
Employment Status			<0.001
- Employed full-time	1 (Reference)		
- Employed part-time	1.17	0.74 - 1.85	
- Retired	1.65	1.26 - 2.17	
- Student	1.32	1.06 - 1.64	
- Unemployed	1.14	0.88 - 1.47	
Geographic Location			0.231
- Urban	1 (Reference)		
- Rural	1.32	0.85 - 2.04	
- Suburban	1.34	0.74 - 1.40	

Table 7 presents findings regarding the cost-effectiveness of endovascular interventions. Participants' perceptions of the interventions' cost-effectiveness were varied, with 11.3% (n=153) believing they were definitely cost-effective and 8.6% (117) believing they were probably cost-effective. Notably, 11.3% (n=153) reported experiencing significant cost savings after the intervention. Satisfaction with the overall value for money was moderately positive (7.3% (n=99) very satisfied and 6.0%(n=81) satisfied).

**Table 7:** Cost-effectiveness of endovascular intervention Participants' perceptions of cost-effectiveness, cost savings, satisfaction, and related factors are summarized in this table. PAD: Peripheral Arterial Disease

		Count	N %
Compared to other treatment options, do you believe that the endovascular intervention was a cost-effective choice for PAD management?	No, probably not	18	1.3%
	Undecided	36	2.6%
	Yes, definitely	153	11.3%
	Yes, probably	117	8.6%
Have you experienced any cost savings related to healthcare visits, medications, or other PAD-related expenses since the endovascular intervention?	No cost savings	27	2.0%
	Yes, moderate cost savings	153	11.3%
	Yes, slight cost savings	18	1.3%
	Yes, significant cost savings	117	8.6%
How satisfied are you with the overall value for money of the endovascular intervention?	Very satisfied	99	7.3%
	Satisfied	81	6.0%
	Dissatisfied	45	3.3%
	Neutral	90	6.6%
Did you experience any unplanned hospital readmissions or emergency room visits after the endovascular intervention?	No	234	17.2%
	Yes	108	7.9%
Are you currently taking fewer medications for PAD management since the endovascular intervention?	No	144	10.6%
	Yes	162	11.9%
How would you rate the financial burden of PAD management before the endovascular intervention?	High	90	6.6%
	Very high	72	5.3%
	Moderate	117	8.6%
	Low	18	1.3%
	Very low	9	0.7%
Has the endovascular intervention reduced your need for additional invasive procedures (e.g., surgery) in managing your PAD?	No reduction	9	0.7%
	Yes, slightly reduced	45	3.3%
	Yes, significantly reduced	90	6.6%
	Yes, moderately reduced	180	13.2%
Are you able to engage in more economic activities (e.g., work, recreational activities) after the endovascular intervention?	No increase in activities	9	0.7%
	Yes, significantly more activities	90	6.6%
	Yes, slightly more activities	81	6.0%
	Yes, moderately more activities	135	9.9%
Has the endovascular intervention contributed to a reduction in your overall healthcare costs?	No reduction	18	1.3%
	Yes, slight reduction	81	6.0%
	Yes, significant reduction	90	6.6%
	Yes, moderate reduction	126	9.3%
How likely are you to recommend endovascular interventions as a cost- effective option for PAD management to other patients?	Unlikely	18	1.3%
	Neutral	99	7.3%
	Very likely	126	9.3%
	Likely	63	4.6%
	Very unlikely	27	2.0%

The factors influencing cost-effectiveness perceptions, as revealed by the multivariate analysis, are depicted in Table 8. Gender significantly influenced cost-effectiveness perceptions (OR = 1.54, 95% CI = 1.20 - 1.98, p < 0.001), as did education level (p < 0.001), with participants holding a master's degree having lower odds of perceiving the interventions as cost-effective (OR = 0.60, 95% CI = 0.42 - 0.85).

**Table 8:** Multivariate (linear regression) model to identify the factors predicting the cost-effectiveness. Odds ratios and confidence intervals are presented for variables impacting perceptions of cost-effectiveness, including gender, education, and employment status.

Variable	Odds Ratio (OR)	95% Confidence Interval (CI)	P-value
Gender (Reference: Female)	1.54	1.20 - 1.98	<0.001
Education level			<0.001
- High school or less	1 (Reference)		
- Diploma	0.92	0.68 - 1.25	
- Bachelor's degree	0.78	0.61 - 1.01	
- Master's degree	0.60	0.42 - 0.85	
Employment Status			<0.001
- Employed full-time	1 (Reference)		
- Employed part-time	1.17	0.74 - 1.85	
- Retired	1.65	1.26 - 2.17	
- Student	1.32	1.06 - 1.64	
- Unemployed	1.14	0.88 - 1.47	
Geographic Location			<0.001
- Urban	1 (Reference)		
- Rural	1.32	0.85 - 2.04	
- Suburban	0.98	0.74 - 1.30	

Our study provides a comprehensive analysis of the effectiveness and cost-effectiveness perceptions of endovascular interventions for managing Peripheral Arterial Disease (PAD) among Saudi patients. The findings underscore the significance of gender, education level, employment status, and geographic location in influencing these perceptions. Participants reported noteworthy improvements in symptom relief, quality of life, and daily activities following endovascular interventions. Additionally, diverse perceptions of cost-effectiveness were observed among participants. These findings contribute crucial insights to the understanding of the efficacy and economic impact of endovascular interventions within the Saudi healthcare landscape, offering valuable guidance for clinical decision-making and resource allocation in PAD management.

## Discussion

Chronic peripheral arterial disease (PAD), characterized by the narrowing or obstruction of arteries supplying blood to the limbs, often leading to pain, reduced function, and a diminished quality of life, is a prevalent condition [1]. This study was conducted in the context of Saudi Arabia, where PAD's prevalence was estimated to be approximately 11.7% (n=56) of 471 patients were recruited. This prevalence is expected to rise significantly as the population ages and accumulates additional risk factors, including cerebrovascular events, coronary artery disease, hypertension, diabetes, smoking, and lipid disorders [3].

Endovascular treatments, such as angioplasty and stenting, have gained popularity as less invasive options for managing PAD, with the potential for improved patient outcomes [12]. However, supporting the adoption of these interventions in the Saudi healthcare system necessitates evidence-based research. Prior studies have explored the effectiveness of endovascular treatments for PAD, with some reporting increased walking distances compared to exercise therapy alone and a reduced incidence of major amputations compared to surgical bypass [13][14]. Nonetheless, these studies had limitations, such as small sample sizes and limited follow-up periods.

This study was designed to address this gap by evaluating the cost-effectiveness of endovascular interventions relative to other treatment modalities in the Saudi healthcare system. It also aimed to assess the effectiveness of these interventions in enhancing clinical outcomes, including symptom relief and quality of life, among Saudi patients with PAD.

The significance of our study lies in its potential to provide valuable insights to healthcare professionals, doctors, and policymakers, enhancing their understanding of how endovascular therapies manage PAD in Saudi patients. This, in turn, could lead to improved patient outcomes. Furthermore, identifying cost-effective management strategies for PAD is essential for optimal resource allocation and alleviating the financial burden associated with the condition.

Our study revealed diverse perceptions regarding the cost-effectiveness of endovascular interventions among participants. A noteworthy portion considered these therapies to be cost-effective. Participants reported varying degrees of symptom relief, including improved ability to complete daily tasks, faster wound healing, and reduced leg cramps or pain during exercise. Additionally, several participants noted an enhancement in overall quality of life.

Gender and education level significantly influenced the perceived effectiveness of these interventions, suggesting the need for a tailored approach. Healthcare providers should carefully consider the choice of endovascular interventions based on individual cases to ensure optimal cost-effectiveness and symptom relief for patients.

Limitations of this study must be acknowledged, including its reliance on retrospective data, potential selection bias, and the absence of long-term follow-up. These limitations may affect the generalizability and robustness of the conclusions, warranting caution in their interpretation. Future research should aim to address these limitations and further explore the nuances of PAD management in the Saudi context.

## Conclusions

In conclusion, based on the questionnaire results, the study demonstrates a positive association between endovascular interventions and improved clinical outcomes in Saudi PAD patients. These findings underscore the effectiveness and cost-efficiency of these interventions, with notable implications for healthcare in Saudi Arabia. To enhance the utilization of endovascular interventions, tailored education, patient empowerment, and shared decision-making are vital, particularly considering gender, education, and geographic factors. Emphasizing positive patient outcomes and addressing cost-effectiveness concerns can foster trust. Healthcare organizations and policymakers can use these insights to optimize PAD management services, improving patient satisfaction and healthcare efficiency in Saudi Arabia.
